# Laparoscopic Management of Benign Splenic Cysts in Children: Partial Splenectomy vs. Deroofing

**DOI:** 10.3390/jcm15041401

**Published:** 2026-02-11

**Authors:** Zenon Pogorelić, Daniel Vidović, Miro Jukić, Zdravko Perko

**Affiliations:** 1 Department of Pediatric Surgery, University Hospital of Split, 21 000 Split, Croatia; 2 Department of Surgery, School of Medicine, University of Split, 21 000 Split, Croatia; 3 Department of Surgery, University Hospital of Split, 21 000 Split, Croatia

**Keywords:** splenic cysts, laparoscopic partial splenectomy, cyst deroofing, spleen-preserving surgery, pediatric surgery, minimally invasive surgery

## Abstract

**Objectives:** This study aimed to compare laparoscopic deroofing and laparoscopic partial splenectomy regarding recurrence, perioperative safety, and short-term postoperative outcomes in pediatric patients. **Methods:** This retrospective single-center study included pediatric patients who underwent laparoscopic partial splenectomy or laparoscopic deroofing for benign splenic cysts between January 2012 and August 2025. Demographics, cyst characteristics, operative variables, postoperative complications, and recurrence were analyzed. The primary outcome was cyst recurrence; secondary outcomes included duration of surgery and length of hospitalization. **Results:** Twenty-three patients met the inclusion criteria: 10 underwent laparoscopic partial splenectomy and 13 laparoscopic deroofing. Groups were comparable in age, sex distribution, cyst diameter, body mass index, and American Society of Anesthesiologists classification (all *p* > 0.3). No conversions to open surgery occurred. Operative time was significantly shorter for partial splenectomy (37.8 ± 7.1 min) compared with deroofing (77.3 ± 33.6 min; *p* = 0.001). Length of hospitalization did not differ significantly between groups (median 2 days; *p* = 0.596). Two minor postoperative complications occurred in the deroofing group, without a significant difference between techniques (*p* = 0.486). A striking difference in recurrence was observed: no recurrences occurred after partial splenectomy (0%), whereas recurrence was documented in 8/13 patients (61.5%) after deroofing (*p* = 0.003). **Conclusions:** Laparoscopic partial splenectomy offers superior long-term efficacy in treating benign splenic cysts in children, with significantly lower recurrence rates and shorter operative times than laparoscopic deroofing. While deroofing remains a technically accessible option, its high recurrence rate limits its role as a definitive treatment. Partial splenectomy appears to be the preferred spleen-preserving technique. Future prospective, multicenter, and ideally randomized studies are warranted to confirm these findings.

## 1. Introduction

Splenic cysts are a rare clinical finding, especially in the pediatric population, with an incidence of about 0.07% [1]. They are usually asymptomatic and can be discovered incidentally during imaging tests or after trauma. Since they are mostly simple, with homogeneous contents and thin walls, imaging is a key diagnostic method [2]. Although uncommon, the most significant clinical sign of splenic cysts is abdominal pain. Sometimes, due to their size, these cysts can cause compressive symptoms, rupture, and hemorrhage [3,4]. Splenic cysts are categorized into parasitic and non-parasitic cysts (NPCs). The primary cause of parasitic cysts is *Echinococcus granulosus* [5]. Over time, NPCs have been classified in various ways; most recently, Morgenstern’s classification has become widely accepted [6].

Because they are rare, splenic cyst treatment mainly depends on expert opinion and case series. In patients with asymptomatic cysts smaller than 5 cm, a watchful waiting approach is often used [4,7,8]. Traditionally, based on clinical experience, cysts larger than 5 cm usually require surgery due to their increased risk of complications, such as rupture and infection, which can lead to hemoperitoneum, abscess formation, and chemical peritonitis [1,4,7,9]. As mentioned earlier, current surgical management depends on cyst size and symptoms [1,4,7,8,9]. Various surgical options for treating splenic cysts have been reported, including complete splenectomy, partial splenectomy, marsupialization of the cyst, and partial cystectomy (unroofing, deroofing, fenestration) [5,7]. For clarity, the term “laparoscopic cyst deroofing” is used throughout this manuscript to describe procedures involving partial excision of the cyst wall without resection of splenic parenchyma.

In the past, the preferred treatment for splenic cysts was open splenectomy [9]. After recognizing the risks of postsplenectomy infections, splenectomy became the preferred method under specific conditions, such as a cyst located in the hilum of the spleen, intrasplenic cysts, cysts inaccessible to fenestration or marsupialization, very large cysts, and polycystic cases [4,5,9,10]. Today, laparoscopic splenectomy is the favored technique if splenectomy is indicated [4]. Given the important role of the spleen and recent technological advances, minimally invasive procedures with spleen-preserving strategies, including laparoscopic deroofing and partial splenectomy, are becoming the preferred options [1,5,7,11]. Laparoscopic deroofing was quickly adopted but tends to have high recurrence rates because the cyst’s epithelial layer cannot be completely removed [7,9,12,13,14,15,16,17,18,19]. It is believed that technical variations do not significantly affect outcomes [12,13,14,15]. In surgical practice, minimally invasive laparoscopic partial splenectomy has emerged as another approach. Although technically challenging and requiring a thorough understanding of regional anatomy, it was rapidly incorporated into surgical protocols with the main aim of removing the cyst, preventing recurrence, and preserving the patient’s immune function by maintaining more than 25% of the splenic tissue [9,11].

The aim of this study is to compare the outcomes of laparoscopic cyst deroofing and laparoscopic partial splenectomy for treating splenic cysts in pediatric patients, focusing on safety, recurrence, and preservation of splenic function to determine the optimal surgical approach for this population.

## 2. Methods

### 2.1. Patients

**Figure 1:**
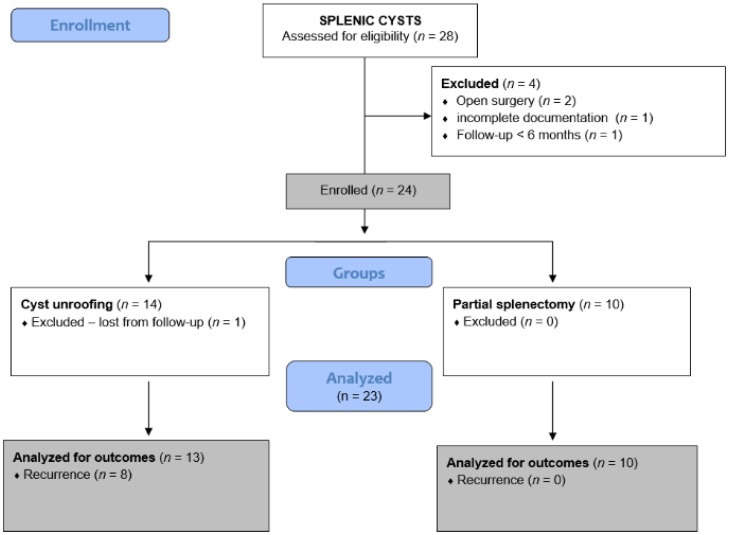
Flow-chart of the study.

This retrospective single-center study included all pediatric patients (0–17 years) who underwent laparoscopic partial splenectomy or laparoscopic cyst deroofing for splenic cysts at the Department of Pediatric Surgery, University Hospital of Split, between 1 January 2012 and 1 August 2025. Eligible patients were treated for symptomatic cysts and/or cysts larger than 5 cm in diameter, with a minimum postoperative follow-up of six months. Patients who underwent open surgery, those with incomplete records, and individuals older than 17 years were excluded from the analysis. During the assessment period, a total of 28 patients who underwent surgery for splenic cysts were identified. However, four participants were excluded from further analysis because they met one or more exclusion criteria. Ultimately, 24 patients met the inclusion criteria and were analyzed further. One patient was subsequently excluded from the comparative analysis due to lack of follow-up data. The flow chart of the study is shown in Figure 1.

### 2.2. Ethical Aspects

This study was conducted in accordance with the ethical standards of the institutional research committee and the 1964 Declaration of Helsinki and its later amendments. The Institutional Review Board approved the protocol prior to data collection (approval number: 520-03/25-01/254; approval date: 30 October 2025). All patient data were anonymized prior to analysis to ensure patient confidentiality. Because this is a retrospective observational study, informed consent was waived or deemed unnecessary in accordance with institutional policy.

### 2.3. Outcomes of the Study

The primary outcome was the recurrence rate between the two groups (cyst deroofing and partial splenectomy). Secondary outcomes were the occurrence of early and late complications, duration of surgery, and length of hospital stay.

### 2.4. Study Design

Information on the following parameters was collected through a comprehensive review of inpatient and outpatient medical records: demographic and clinical details (age, sex, height, weight, body mass index [BMI], and American Society of Anesthesiologists (ASA) physical status classification), cyst features (diameter, location, and symptoms), laboratory results (complete blood count, serology, and inflammatory markers), imaging studies (ultrasound, computed tomography (CT), magnetic resonance imaging (MRI), and contrast-enhanced imaging), histopathological findings, duration of surgery, length of hospital stay, and recurrence. Postoperative complications were assessed during follow-up.

Patients were divided into two groups based on the type of surgical procedure: laparoscopic partial splenectomy (*n* = 10) and laparoscopic deroofing (*n* = 13). Indications for surgery included abdominal pain, fullness or compression, cysts larger than 5 cm in diameter, and cyst growth during follow-up. Surgery was also performed for complicated cysts (rupture, infection, or hemorrhage) or cysts in areas at higher risk of rupture.

The choice of surgical technique (partial splenectomy or cyst deroofing) depended on the cyst’s anatomical location, proximity to the splenic hilum, the amount of preserved splenic tissue, and the surgeon’s preference and experience. Partial splenectomy was preferred for deeply located or recurrent cysts when adequate resection margins could be achieved while preserving sufficient splenic tissue. Deroofing was performed for superficially located cysts with accessible walls and no hilar involvement.

### 2.5. Surgical Technique

In all cases, the patient was positioned supine with a small roll or bump under the left hemithorax to achieve a slight left-side-up tilt, combined with gentle reverse Trendelenburg positioning to enhance exposure of the left upper quadrant. A standard laparoscopic approach was used. Pneumoperitoneum was established with a Veress needle at the umbilicus, and intra-abdominal pressure was maintained between 8 and 12 mmHg, depending on the patient’s age and height. Usually, three trocars were used; a fourth was added only if necessary for retraction or improved exposure. The trocar placements were as follows: a 10 mm trocar was inserted supraumbilically for the 30° laparoscope; a 12 mm main working trocar was placed in the left midclavicular line, approximately 4–6 cm below the costal margin; a 5 mm trocar was positioned in the left anterior axillary or subcostal line for retraction and exposure of the hilum or nearby tissues. If needed, especially with a large spleen or difficult exposure, a fourth 5 mm trocar was inserted more laterally, such as along the left posterior axillary line or just lateral to the tip of the 11th rib, to assist with retraction. Occasionally, trocar positions were slightly adjusted intraoperatively based on spleen size, patient habitus, and surgical exposure requirements. The surgeon stood on the patient’s right side, the assistant was positioned between the patient’s legs, and the scrub nurse was on the left. The monitor was located on the patient’s left side. All procedures were performed by experienced pediatric surgeons with expertise in minimally invasive splenic surgery.

#### 2.5.1. Laparoscopic Cyst Deroofing

**Figure 2:**
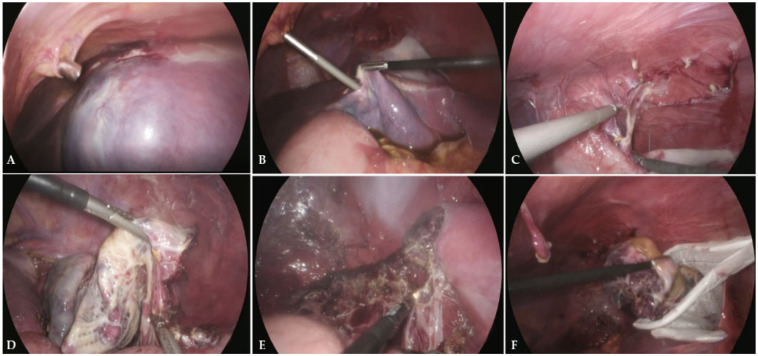
Laparoscopic cyst deroofing in a 16-year-old female using a harmonic scalpel for a cyst at the upper pole of the spleen (20 × 17 × 15 cm). (**A**) Identifying the splenic cyst; (**B**) controlled decompression and aspiration of fluid; (**C**) dissecting the cyst wall from the diaphragm and surrounding tissues; (**D**) partially excising the cyst wall with a harmonic scalpel; (**E**) inspecting, irrigating, and cauterizing the cyst cavity; (**F**) removing the excised cyst wall with an endoscopic retrieval bag.

For laparoscopic cyst deroofing, the cyst was identified (Figure 2A), decompressed (Figure 2B), and carefully dissected from surrounding tissue and splenic parenchyma using both blunt and sharp dissection (Figure 2C). The cyst wall was partially excised with an ultrasonic scalpel (Ultracision™; Ethicon Endo-Surgery, Cincinnati, OH, USA) to achieve wide deroofing while preserving the underlying splenic tissue (Figure 2D). The cyst cavity was inspected for bleeding, irrigated with saline, and, in most cases, cauterized to destroy epithelial remnants and reduce the risk of recurrence. An omentopexy was then performed by placing a segment of omentum into the cystic cavity to promote drainage and prevent fluid reaccumulation (Figure 2E). The excised cyst wall was retrieved using an endoscopic retrieval bag (Ecosac EMP 70; Espiner Medical Ltd., Measham, UK) (Figure 2F). The excised specimen was sent for pathologic examination.

#### 2.5.2. Laparoscopic Partial Splenectomy

**Figure 3:**
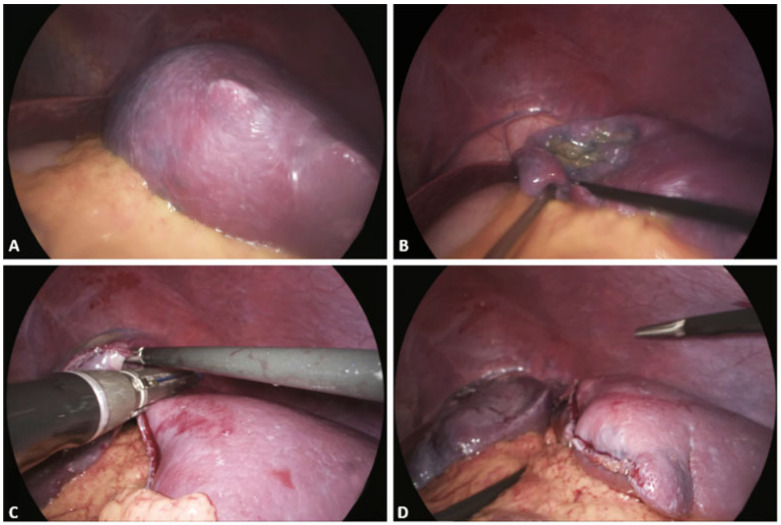
Partial laparoscopic splenectomy in a 14-year-old patient using a linear endostapler for a large cyst at the upper pole of the spleen (10 × 8 × 6 cm). (**A**) Inspecting the abdominal cavity and locating the splenic cyst; (**B**) safely decompressing and aspirating the cystic fluid; (**C**) transecting the parenchyma with a linear stapler about 1 cm from the cyst edge into healthy splenic tissue; (**D**) examining the remaining spleen to ensure proper perfusion and proper control of bleeding.

After inspecting the abdominal cavity and identifying the cyst (Figure 3A), the cyst was first punctured and decompressed (Figure 3B), then carefully dissected from surrounding tissues using a harmonic scalpel (Ultracision™; Ethicon Endo-surgery, Cincinnati, OH, USA). The segmental anatomy of the spleen was identified intraoperatively by direct visualization. After decompression and cyst dissection, parenchymal resection was performed using a linear stapler, initially the Echelon Flex™ (Ethicon, Cincinnati, OH, USA) with blue cartridges (Figure 3C), and later the Signia™ Stapling System (Medtronic, Minneapolis, MN, USA) with purple cartridges, approximately 1 cm from the cyst margin into healthy splenic tissue, ensuring preservation of the remaining spleen’s vascular supply. Hemostasis was carefully secured. The resected specimen was placed in an endoscopic retrieval bag (Ecosac EMP 70; Espiner Medical Ltd., Measham, UK) and removed through the 12 mm port. The remaining splenic tissue was examined to confirm adequate perfusion (Figure 3D). The excised specimen was sent for pathological examination.

### 2.6. Postoperative Protocol and Follow-Up

All procedures were performed as minimally invasive, spleen-preserving surgeries. In our institution, pediatric patients undergoing splenic surgery are routinely monitored in the intensive care unit for the first 24 postoperative hours as part of a standardized postoperative observation protocol. This unit primarily serves a monitoring function rather than intensive treatment, and no patient required organ support or intensive therapeutic interventions. Postoperative recovery was generally smooth. During the first 24 h after surgery, all patients were monitored in the intensive care unit to allow close observation of vital signs and early detection of potential complications. Patients were discharged within 2–3 days if they were afebrile, hemodynamically stable, tolerated oral intake, had adequate pain control with oral analgesics, and showed no signs of surgical complications. Oral intake was resumed 4–8 h after surgery, depending on recovery from anesthesia and the absence of nausea or vomiting. For pain management, non-opioid analgesics were administered as needed. Paracetamol (Paracetamol Kabi, Fresenius Kabi, Zagreb, Croatia) was given at a dose of 15 mg/kg, and ibuprofen (Brufen, Mylan, Zagreb, Croatia) at 10 mg/kg per dose. After discharge, patients were followed up at the outpatient clinic. Skin sutures were removed on postoperative day 7. Follow-up ultrasonography was performed 6 months after surgery to check for cyst recurrence or residual lesions, followed by annual clinical and ultrasonographic evaluations.

### 2.7. Statistical Analysis

Continuous variables were presented as mean ± standard deviation (SD) if normally distributed or as median and interquartile range (IQR) if not normally distributed. Categorical variables were presented as absolute numbers and percentages. Normality was assessed using the Shapiro–Wilk test. For group comparisons, the independent samples *t*-test was used for normally distributed continuous variables, and the Mann–Whitney U test was used for non-normal data. Categorical variables were compared using either the chi-square test or Fisher’s exact test, as appropriate. Statistical analyses were conducted using Python (version 3.11; Python Software Foundation, https://www.python.org, accessed on 2 December 2025). Data were visualized using standard Python plotting tools.

## 3. Results

A total of 23 patients were included in the study, of whom 10 (43.48%) underwent laparoscopic partial splenectomy, and 13 (56.52%) underwent laparoscopic cyst deroofing. The mean age was similar in both groups (15 years), with a predominance of female patients. No significant differences were found between the groups in height (*p* = 1.000), weight (*p* = 0.836), or BMI (*p* = 0.309). Most patients were classified as ASA group I. Both groups were comparable in demographic and preoperative characteristics, with no statistically significant differences. Additionally, the groups were similar and nearly symmetrical in cyst characteristics (Table 1). Serological testing for *Echinococcus* spp. was negative in all patients.

**Figure 4:**
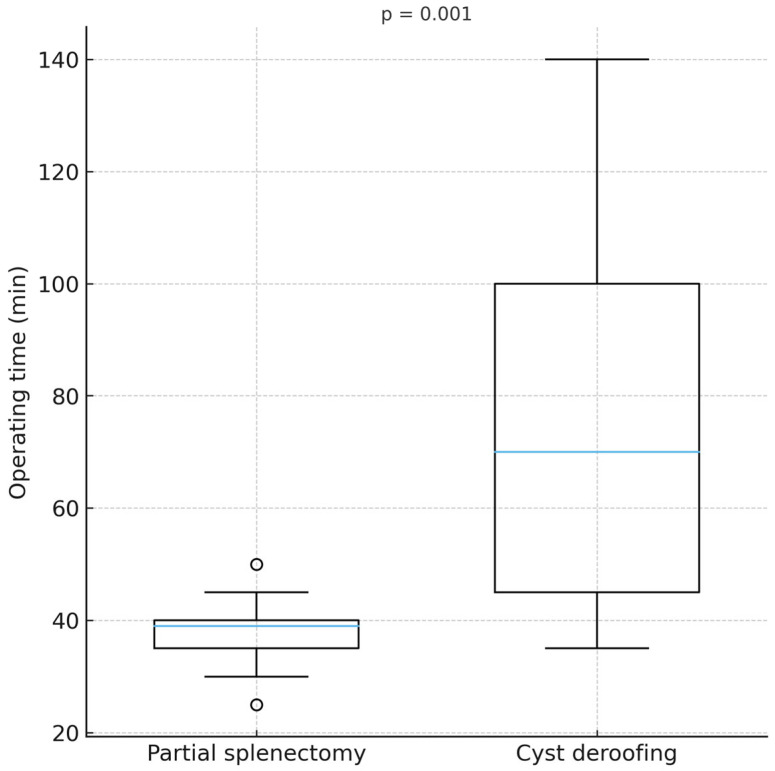
Comparison of surgery duration between the studied groups.

No intraoperative conversions to open surgery occurred, and estimated blood loss was minimal in all cases (below 50 mL). The duration of the surgical procedure (37.8 ± 7.1 min versus 77.3 ± 33.6 min, *p* = 0.001) was significantly shorter in the laparoscopic partial splenectomy group than in the deroofing group, as shown in Figure 4. There was no statistically significant difference in the length of hospital stay or postoperative complications, although 2 (15.4%) minor complications were recorded in the deroofing group: one surgical site infection treated with local incision and drainage, and one postoperative wound hematoma that resolved with conservative management. Neither complication required reoperation or resulted in prolonged hospital stay.

Follow-up duration differed significantly between the groups, with longer follow-up observed in the deroofing group (69.08 ± 41.36 months) compared to the partial splenectomy group (24.90 ± 16.69 months, *p* = 0.003).

**Figure 5:**
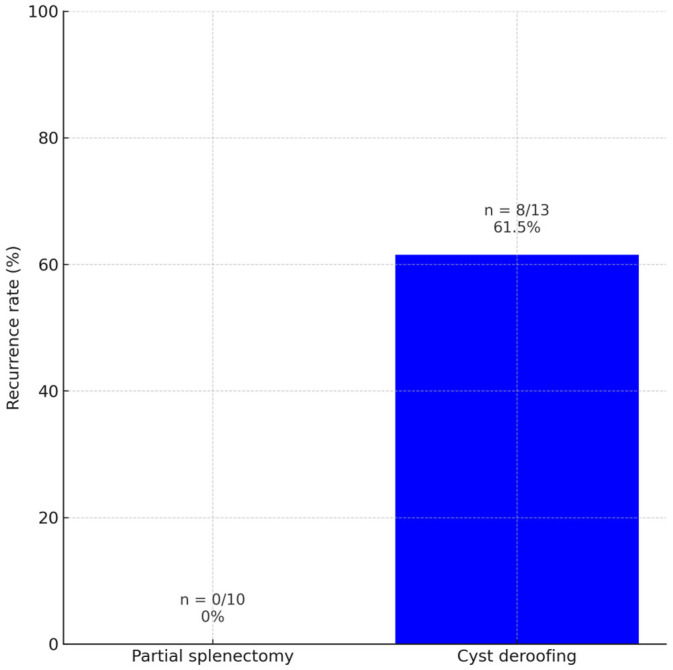
Comparison of recurrence rates between the studied groups.

A statistically significant difference was observed in the recurrence of splenic cysts. No recurrence was found in the partial splenectomy group (0%), whereas recurrence occurred in 8 patients (61.5%) in the deroofing group (*p* = 0.003) (Figure 5). The most common histopathological finding in both groups was epithelial cyst (Table 2).

## 4. Discussion

This retrospective single-center study in a pediatric population compared two spleen-preserving laparoscopic techniques, partial splenectomy and cyst deroofing, for the treatment of nonparasitic splenic cysts, with particular emphasis on recurrence and long-term effectiveness. The most important finding of this study is the complete absence of recurrence after laparoscopic partial splenectomy, in contrast to the high recurrence rate observed after laparoscopic cyst deroofing. This finding is the central message of our experience and supports laparoscopic partial splenectomy as the most definitive spleen-preserving surgical option for pediatric splenic cysts when anatomically feasible.

Both groups were comparable for demographic characteristics, cyst size, localization, clinical presentation, and histopathological findings, indicating that outcome differences were primarily related to surgical technique rather than baseline patient or cyst-related factors. No intraoperative conversions to open surgery occurred, and blood loss was minimal in all cases, confirming the safety of both laparoscopic approaches in experienced centers. Operative time was significantly shorter in the partial splenectomy group, despite the technically more demanding nature of this procedure. This finding may appear counterintuitive; however, it likely reflects increasing institutional experience over time, a well-established learning curve, and the use of a standardized stapler-based resection technique. In our practice, laparoscopic partial splenectomy follows clearly defined operative steps, allowing efficient parenchymal transection and reliable hemostasis. In contrast, cyst deroofing often requires more extensive dissection of the cyst wall from surrounding structures and meticulous management of the residual cavity, which may prolong operative time despite its perceived technical simplicity.

Postoperative recovery was similar in both groups, with no significant difference in length of hospital stay. However, postoperative complications and all documented recurrences occurred exclusively in patients who underwent cyst deroofing. These findings further emphasize that although cyst deroofing is technically simpler and initially appealing, it may be associated with inferior long-term outcomes due to incomplete removal of the cyst epithelium. Procedures that leave residual epithelial lining are inherently prone to recurrence, regardless of technical modifications such as cauterization or omentopexy.

The difference in follow-up duration between the groups must be acknowledged, as the cyst deroofing cohort had a significantly longer follow-up period. Although this may have increased the likelihood of detecting recurrences in that group, the markedly high recurrence rate observed and the absence of recurrence in the partial splenectomy group, even with a shorter follow-up period, remain clinically relevant and support the durability of this approach.

Comparison with previously published studies reveals both concordant and divergent findings. The median age of patients in our study was higher than that reported by Karaaslan and Czauderna et al., who included younger pediatric populations and used mixed open and laparoscopic techniques [13,14]. These differences are likely attributable to variations in inclusion criteria, referral patterns, and institutional practice. In contrast, the age and cyst size of patients in our cohort closely resemble those reported by Hassoun et al., who also described predominantly older children with large, symptomatic cysts requiring surgical intervention [7].

Mean cyst diameter in our study exceeded 13 cm in both groups, consistent with the literature indicating that larger cysts are more likely to become symptomatic and require operative management. While Karaaslan reported slightly smaller cysts, Hassoun et al. documented cyst sizes comparable to those in our series [7,13]. These findings suggest that cyst size alone does not dictate the optimal surgical approach; rather, cyst location, depth, and relationship to splenic vasculature play a decisive role in technique selection.

Recurrence remains the most critical outcome measure when evaluating spleen-preserving procedures. Several studies have shown that laparoscopic cyst excision, fenestration, or deroofing is associated with high recurrence rates, primarily due to incomplete removal of the cyst wall [12,13,14,18]. Schier et al. reported particularly unfavorable outcomes following laparoscopic deroofing, concluding that technical modifications alone are insufficient to prevent recurrence when epithelial remnants remain in situ [12]. Similarly, a large European multicenter study confirmed significantly higher recurrence rates after fenestration and cyst excision compared with partial splenectomy, underscoring the importance of definitive cyst removal [14]. Recent pediatric data further support these observations. Santángelo et al. reported favorable short- and mid-term outcomes of minimally invasive spleen-preserving surgery in children, emphasizing that procedures leaving residual epithelial lining, such as deroofing or fenestration, are associated with a higher risk of recurrence, whereas more radical laparoscopic approaches reduce recurrence rates [20].

Systematic reviews and retrospective analyses further support these observations. Sinha and Agrawal reported recurrence rates of up to 41% after laparoscopic procedures, with most recurrences occurring after deroofing or partial decapsulation [15]. In contrast, partial splenectomy and total splenectomy were associated with substantially lower recurrence rates. Although Karaaslan reported a low overall recurrence rate, the majority of laparoscopic procedures in that study consisted of cystectomy rather than partial splenectomy, with only one patient undergoing partial splenectomy [13].

In our series, laparoscopic partial splenectomy resulted in no recurrences, whereas cyst deroofing was associated with a recurrence rate exceeding 60%, reinforcing the superiority of partial splenectomy for long-term disease control. In cases of cyst recurrence, our management strategy is individualized. Small, asymptomatic recurrent cysts are managed with close ultrasonographic follow-up, whereas symptomatic or enlarging recurrences are preferentially treated with laparoscopic partial splenectomy when anatomically feasible. Repeat cyst deroofing is generally avoided due to the high risk of further recurrence.

Alternative minimally invasive approaches, such as percutaneous aspiration with or without sclerotherapy, have also been described; however, these techniques are associated with high rates of persistence and recurrence and are therefore not considered definitive treatment for large or symptomatic splenic cysts [7,18,19]. Their role remains limited to selected cases or to temporary symptom relief.

Although this study focused on spleen-preserving laparoscopic techniques, total splenectomy remains necessary in selected cases, including very large cysts, polycystic disease, hilar involvement, or cysts inaccessible to partial resection or deroofing [4,5,15]. Several studies have reported excellent short-term outcomes and no recurrence after total splenectomy [7,13,15]. Recent evidence suggests that cyst size alone should not be considered an absolute indication for total splenectomy. Aoun et al. demonstrated that even giant splenic cysts can be successfully managed with minimally invasive techniques, provided adequate surgical expertise and careful patient selection are ensured [21]. However, the well-documented risks of overwhelming post-splenectomy infection, increased susceptibility to sepsis, and long-term mortality make spleen preservation particularly important in pediatric patients [4,10,11,16]. Although splenic immune function was not directly assessed in this study, preservation of sufficient splenic parenchyma is widely accepted as a surrogate indicator of maintained immunological competence in pediatric patients.

The indication for surgical intervention in nonparasitic splenic cysts remains a subject of ongoing debate. Many authors advocate surgery for symptomatic cysts or cysts larger than 5 cm because of the risk of rupture, hemorrhage, and infection [4,7,11,15]. Conversely, Di Lena et al. reported favorable outcomes with a watchful waiting strategy in selected asymptomatic patients, even with large cysts [17]. These divergent strategies underscore the absence of a universally accepted treatment algorithm and emphasize the need for individualized decision-making based on cyst characteristics, patient symptoms, and institutional expertise.

From a technical perspective, laparoscopic surgery offers clear advantages over open surgery, including reduced postoperative pain, faster recovery, and a shorter hospital stay [22,23,24]. Recent reviews support the laparoscopic approach for splenic cysts but emphasize that long-term success depends on selecting the appropriate technique and surgical experience, with partial splenectomy providing the most reliable balance between spleen preservation and recurrence prevention [16,24]. It should be acknowledged that laparoscopic partial splenectomy is a technically demanding procedure, and the favorable outcomes reported in this study reflect the experience of a high-volume center with expertise in advanced pediatric minimally invasive surgery. Consequently, the applicability of these results to lower-volume or less experienced institutions may be limited. Wider adoption of this technique should be supported by structured training, an appropriate learning curve, and careful patient selection, with referral to specialized centers for complex cases.

Several limitations of this study should be acknowledged. Its retrospective design introduces potential selection bias and limits control over confounding variables. The retrospective and non-randomized design of the study, with the choice of surgical technique partly influenced by anatomical considerations and surgeon preference, represents a potential source of selection bias that should be considered when interpreting the results. The rarity of nonparasitic splenic cysts resulted in a relatively small sample collected over an extended period, which may affect external validity and limit statistical power, particularly for secondary outcomes, increasing the risk of type II error. Additionally, this was a single-center study, and outcomes may differ in institutions with less experience in advanced laparoscopic splenic surgery. Finally, a relevant limitation of this study is the significantly longer follow-up in the cyst deroofing group compared with the partial splenectomy group, which may have increased the likelihood of detecting recurrences in that cohort. Conversely, the shorter follow-up in the partial splenectomy group limits the ability to completely exclude very late recurrences. However, the high recurrence rate observed after deroofing and the absence of recurrence after partial splenectomy, even within a shorter follow-up period, suggest a clinically meaningful difference between the two techniques.

Future multicenter prospective studies with larger cohorts and standardized surgical protocols are needed to confirm these findings. A longer follow-up would further clarify long-term outcomes and help establish evidence-based guidelines for the optimal surgical management of pediatric splenic cysts.

## 5. Conclusions

The present study demonstrates that, in pediatric patients with benign splenic cysts, laparoscopic partial splenectomy provides a durable spleen-preserving solution, with excellent long-term outcomes and no observed recurrences. In contrast, laparoscopic cyst deroofing, although safe and minimally invasive, is associated with an unacceptably high recurrence rate, limiting its role as a definitive treatment. These findings suggest that, whenever anatomically feasible and performed in experienced centers, laparoscopic partial splenectomy should be considered the preferred surgical strategy for large or symptomatic splenic cysts in children. Individualized treatment planning remains essential, taking into account cyst location, splenic vascular anatomy, and surgeon expertise.

## Figures and Tables

**Table 1:** Preoperative and demographic data of subjects and splenic cyst features.

Variable	Partial Splenectomy (*n* = 10)	Cyst Deroofing (*n* = 13)	*p*
Demographic and baseline characteristics of the patients			
Age (years)	15.55 ± 1.57	15.12 ± 2.07	0.573 ^t^
Female gender	7 (70.0%)	9 (69.2%)	1.000 ^f^
Height (cm)	175.00 (163.50–177.00)	175.00 (162.00–177.00)	1.000 ^u^
Weight (kg)	62.60 ± 15.09	64.00 ± 16.74	0.836 ^t^
BMI (kg/m^2^)	18.1 ± 3.5	20.1 ± 4.9	0.309 ^t^
ASA I ASA II	9 (90.0%) 1 (10.0%)	12 (92.3%) 1 (7.7%)	1.000 ^f^
Cyst’s characteristics			
Cyst diameter (cm)	14.10 ± 5.67	13.85 ± 4.61	0.910 ^t^
Localization: Lower pole	2 (20.0%)	3 (23.1%)	1.000 ^f^
Upper pole	8 (80.0%)	10 (76.9%)	
Symptomatic presentation	8 (80.0%)	11 (84.6%)	1.000 ^f^

Continuous variables are presented as mean ± standard deviation or median (Interquartile range). Categorical variables are presented as absolute numbers and percentages. Abbreviations: BMI—Body mass index; ASA—American Society of Anesthesiologists. ^t^ Welch’s *t*–test, ^u^ Mann–Whitney U, ^f^ Fisher’s exact.

**Table 2:** Treatment outcomes and histopathological reports.

Variable	Partial Splenectomy (*n* = 10)	Cyst Deroofing (*n* = 13)	*p*
Outcomes of treatment			
Duration of surgery (min)	37.80 ± 7.11	77.31 ± 33.64	0.001 ^t^
Length of hospitalization (days)	2.00 (2.00–2.00)	2.00 (2.00–3.00)	0.596 ^u^
Postoperative complications (any)	0 (0.0%)	2 (15.4%)	0.486 ^f^
Recurrence	0 (0.0%)	8 (61.5%)	0.003 ^f^
Follow-up (months)	24.90 ± 16.69	69.08 ± 41.36	0.003 ^t^
Histopathological findings			
Dermoid cyst	1 (10.0%)	0 (0.0%)	0.435 ^f^
Epithelial cyst	8 (80.0%)	12 (92.3%)	0.560 ^f^
Pseudocyst	1 (10.0%)	1 (7.7%)	1.000 ^f^

Continuous variables are presented as mean ± standard deviation or median (interquartile range). Categorical variables are presented as absolute numbers and percentages. ^t^ Welch’s *t*–test, ^u^ Mann–Whitney U, ^f^ Fisher’s exact test.

## Data Availability

The data assessed and reported here can be obtained from the authors upon reasonable request and following ethical and privacy principles.
